# Prevalence, subtypes, and risk factors of adverse childhood experiences among Chinese residents: a multicenter cross-sectional study

**DOI:** 10.3389/fpubh.2024.1453517

**Published:** 2024-10-02

**Authors:** Yinhai Chen, Yuanwei Lu, Xiuying Wen, Tong Zhou, Xiong Ke

**Affiliations:** ^1^School of Nursing, North Sichuan Medical College, Nanchong, China; ^2^School of Public Health, North Sichuan Medical College, Nanchong, China; ^3^Key Laboratory of Digital-Intelligent Disease Surveillance and Health Governance, North Sichuan Medical College, Nanchong, China; ^4^Sichuan Primary Health Care Research Center, North Sichuan Medical College, Nanchong, China

**Keywords:** ACEs, latent class analysis, anxiety, depression, cross-sectional study, clustering

## Abstract

**Background:**

Addressing the long-term physical and mental health impacts of adverse childhood experiences (ACEs) remains a significant public health challenge. Additionally, ACEs can contribute to intergenerational transmission, affecting future generations. While previous studies have primarily focused on children and adolescents, there is limited data on ACE subtypes and influencing factors among the general adult population, particularly in China. This study aims to explore the prevalence, subtypes, and factors influencing ACEs among Chinese adults.

**Method:**

A total of 1,932 Chinese residents from southwest China (Sichuan, Yunnan, Guizhou provinces, and Chongqing Municipality) participated in the study, consisting of 867 men (44.9%) and 1,065 women (55.1%). Latent class analysis (LCA) was used to identify ACE clusters, and regression analysis examined associations between ACE clusters and demographic factors, physical illness, and mental health outcomes.

**Results:**

The findings revealed that 28.7% of participants had experienced at least one ACE, while 13.2% had experienced three or more ACEs. Three distinct ACE clusters were identified: a low ACE group, a high emotional and physical abuse/family dysfunction group, and a high ACE/sexual abuse group. Regression analysis showed significant associations between childhood adversity and demographic factors (age, education, birthplace), as well as physical and mental health outcomes (anxiety, depression). ANOVA further confirmed significant differences in depression and anxiety scores across the clusters.

**Conclusion:**

These findings offer critical insights for developing targeted public health interventions. Policymakers should consider strategies to reduce childhood ACEs and mitigate their long-term consequences, with particular attention to high-risk groups.

## 1 Introduction

Adverse childhood experiences (ACEs) are potentially traumatic events that occur during childhood (before the age of 18 years) (1). Various studies have shown that many adults experience varying degrees of ACEs before the age of 18 years. A meta-analysis showed that the combined prevalence of individuals with one ACE in Europe was 23.5%; this was 18% with two or more ACEs. The combined prevalence of individuals with one ACE in North America was 23.4, and 35.0% with two or more ACEs (2). Similarly, a study in Iceland found an ACE prevalence of 80.3% (3). Research in China showed that more than 35% of college students reported experiencing at least one type of childhood maltreatment (4). Numerous studies have also shown a strong relationship between ACEs and subsequent physical and mental health. A meta-analysis concluded that individuals with ACEs or with ≥4 ACEs had an increased risk of developing diabetes in adulthood compared with individuals without ACEs (5). ACEs are also a risk factor for anxiety, depression (6), cancer (7), obesity (8), HIV infection (9), and post-traumatic stress disorder (10). The high individual prevalence of ACEs and subsequent serious adverse effects have contributed to an increase in the number of reports of ACEs over the past 20 years (11). However, due to individual heterogeneity and regional differences, how to effectively prevent and mitigate ACEs remains a challenge, especially in middle- and lower-income countries (12). One particular area of research, the co-occurrence of ACEs, may play a key role in effective interventions to prevent ACEs.

Identifying groups of children with similar experiences through latent class analysis (LCA) will facilitate better intervention (13). An individual may have more than one ACE, and subgroups with similar experiences may benefit from similar interventions (14). Some studies have also shown that different potential clusters of ACEs have inconsistent negative effects in childhood or adulthood (15, 16). Previous reports have generally focused on the association between ACEs and physical and mental health, as well as negative behaviors in certain individuals, to suggest measures for improvement (3, 17, 18). However, a key opportunity may have been overlooked in that it might be better to target specific clusters of ACEs in developing public health strategies. Identifying the co-occurrence of ACEs in low- and middle-income countries is therefore critical for policymakers.

In recent years, many researchers have begun to examine the co-occurrence of ACEs (13, 19, 20). For example, a study in the United States identified four clusters of ACEs: high adversity, low adversity, child abuse, and parental substance use (21). Four ACE clusters were identified in a study of preschoolers: separation, parental incarceration, family dysfunction, and child abuse (22). Another study of adolescents in China identified three clusters of ACEs: low adversity, moderate adversity, and high adversity (23). Identifying groups of children with similar experiences through latent class analysis (LCA) will facilitate better intervention (13). Some studies have also shown that different potential clusters of ACEs have inconsistent negative effects in childhood or adulthood (15, 16). Previous reports have generally focused on the association between ACEs and physical and mental health, as well as negative behaviors in certain individuals, to suggest measures for improvement (3, 17, 18). Despite these advances, research in this area has been limited, particularly regarding the general Chinese population (24).

As far as we know, cluster analysis of ACEs in the general residents is still lacking in China. Most existing studies in China have focused on samples of college students and adolescents (23, 25–30), leaving a gap in understanding ACEs in the general population. Given the potential lifelong impact of ACEs on health and the possibility of intergenerational transmission (31), it is essential to examine ACE prevalence and clustering in the general Chinese population to inform public health strategies.

Specifically, the objectives of this study are: (1)To investigate the prevalence of ACEs among Chinese residents and examine whether distinct clusters of ACEs are present in the general population of China; (2)To describe the associations between different ACE clusters and demographic factors, and explore their relationships with physical and mental health; (3)To explore whether there are significant differences in depression and anxiety scores across different ACE clusters, providing insights for intervention strategies.

## 2 Methods

### 2.1 Research design and population

This study was conducted between July 20, 2023, and August 31, 2023, in Southwest China, covering Sichuan, Yunnan, Guizhou provinces, and Chongqing Municipality. We used non-probability stratified sampling across multiple levels, including provincial, municipal, district/county, town/street, and community levels. At the individual level, quota sampling was based on gender and age, maintaining a 1:1 gender ratio and ensuring the age distribution closely aligned with China’s “population pyramid.” The research team, composed of research assistants and students, collaborated with local community health centers and resident committees to recruit participants. Once enough individuals expressed willingness to participate, questionnaires were distributed, either electronically or on paper, via WeChat QR codes. A total of 2,270 individuals were recruited, of which 1,979 responded, yielding an 87.2% response rate. After excluding 47 incomplete questionnaires, 1,932 valid responses were retained, resulting in a 97.6% qualification rate. Inclusion criteria were (1) age ≥ 18 years, (2) Chinese nationality, (3) Chinese permanent resident (annual travel time ≤ 1 month), (4) ability to complete the questionnaire independently or with the help of an investigator, and (5) able to understand the meaning of each item on the questionnaire. Exclusion criteria were (1) delirious or abnormal consciousness, (2) cognitive dysfunction, and (3) unwilling to participate in this study. This study was approved by the ethical review board of Shandong Provincial Hospital (SWYX: No. 2023–198). We obtained the written consent of all participants.

### 2.2 Measures

#### 2.2.1 Demographic data

Demographic variables were collected including sex, age, education, birthplace, and physical disease. Age was divided into four groups: 18–30, 31–45, 46–60, and 61–90 years. Because all participants were ordinary residents, with nearly half of the participants being middle-aged and older adults, we did not subdivide participants according to education level but established two categories for education level: less than high school and more than high school education. As for physical diseases, we mainly asked whether participants had any chronic physical diseases and the specific types of diseases.

#### 2.2.2 ACE scale

The ACE scale was developed based on previous World Health Organization surveys in multiple countries (32). We captured 17 ACE indicators and grouped them into seven categories, namely emotional abuse (A1, A2), physical abuse (A3, A4), sexual abuse (A5–A8), household substance abuse (A9, A10), household mental illness (A11, A12), household incarceration (A13), domestic violence (A14), and parental separation/divorce/death (A15–A17) (33, 34). Each ACE indicator was further dichotomized (0: no, 1: yes). The number of ACEs was summed to obtain the cumulative score.

#### 2.2.3 Patient health questionnaire-9

The PHQ-9 has been recognized worldwide as a reliable tool in screening for depression (35). The questionnaire consists of nine items and uses the Richter Level 4 scale. The scoring criteria are 0–4: normal, 5–9: mild depression, 10–14: moderate depression, 15–21: moderate to severe depression, and 22–27: severe depression. In this study, Cronbach’s alpha was 0.937.

#### 2.2.4 The 7-item generalized anxiety disorder questionnaire

GAD-7 is a convenient and reliable screening tool for anxiety (36). The scale consists of seven items. The scoring criteria are 0–4: normal, 5–9: mild anxiety, 10–14: moderate anxiety, and 15–21: severe anxiety. It is generally considered that the optimal cut point is ≥10 (37). In this study, Cronbach’s alpha was 0.947.

#### 2.2.5 Statistical analysis

Mpuls 8.3 was used to estimate the latent class, and five class models were initially identified using 17 ACE indicators. Latent class analysis (LCA) was selected because it allows for the identification of subgroups with similar patterns of ACEs, which is essential for understanding how different ACE clusters relate to health outcomes. The optimal model was determined based on model fit criteria, including the Akaike information criterion (AIC), Bayesian information criterion (BIC), adjusted Bayesian information criterion (aBIC), entropy, and the bootstrap likelihood ratio test (BLRT). Specifically, smaller AIC, BIC, and aBIC values indicated better model fit, while entropy values closer to 1 suggested higher classification accuracy, with entropy >0.80 generally indicating classification accuracy above 90%. Additionally, *p*-values <0.05 for the LMRT and BLRT suggested that the k-class model provided a better fit than the k-1 class model. After determining the optimal model based on these criteria, SPSS 27.0 was used for further statistical analysis. The chi-square test was used for comparisons between groups. Multinomial Logistic regression was used with demographic characteristics and health status as independent variables, and the LCA classification results as dependent variables, to assess associations between the latent classes and health outcomes. We further compared the anxiety and depression scores of different ACE clusters using analysis of variance (ANOVA). A *p*-value <0.05 was considered statistically significant.

## 3 Results

### 3.1 Participant characteristics

A total of 1932 participants were included in the study, including 867 men (44.9%) and 1,065 women (55.1%). The distribution of participants in different age groups was basically balanced, and the largest age group was 18–30 years (31.2%). More than half of the participants had no post-secondary education (58.7%). A total of 141 (7.3%) participants had hypertension and 470 (27.3%) had other diseases (urinary, respiratory, digestive, and neurological diseases). In total, 28.7% of participants had at least one type of ACE, and 13.2% of residents had three or more ACEs. The prevalence of depression and anxiety was 53.9 and 45.2%, respectively. Details are shown in Table 1.

**Table 1:** Participant characteristics.

Demographics		n	%
Sex	Male	867	44.9
	Female	1,065	55.1
Age (years)	18–30	606	31.2
	31–45	468	24.1
	46–60	472	24.3
	61–90	386	19.9
Education	High school or less	1,135	58.7
	More than high school	797	41.3
Birthplace	Rural	1,175	60.8
	Urban	757	39.2
Health status		n	%
Disease	No	1,321	68.4
	Hypertension	141	7.3
	Other diseases	470	24.3
Depression	No	890	46.1
	Mild depression	623	32.2
	Moderate or severe depression	419	21.7
Anxiety	No	1,060	54.9
	Mild anxiety	575	29.8
	Moderate or severe anxiety	297	15.4
No. of ACEs	0	1,377	71.3
	1	172	8.9
	2	128	6.6
	3+	255	13.2

### 3.2 LCA results

We preliminarily identified one to five latent clusters. Table 2 shows that as the number of classes increased, the AIC, BIC, and aBIC indexes gradually decreased; all entropy values were > 0.9. This implies that no matter which model we ultimately chose, participants would be assigned to a cluster with high accuracy. With a *p*-value of the k-class model <0.05, the fitting degree of the model was better than that of the k-1 class model. Table 2 shows that the p-value of C2-C4 was <0.05. However, in C4, two of the four clusters had a small population proportion (1.3 and 2.2%). In C3, although the proportion of one cluster was 3%, the prevalence of 3% for the entire Chinese population is substantial because the prevalence of ACEs in our sample was less than 30%. Therefore, we finally chose C3, which includes three potential clusters: Class 1 (C1, 81.2%), Class 2 (C2, 15.8%), and Class 3 (C3, 3%). Details are shown in Table 2.

**Table 2:** Model fitting statistics.

	AIC	BIC	aBIC	Entropy	LMR-LRT	BLRT	Class size (%)
C1	12136.567	12231.194	12177.185				
C2	9592.999	9787.82	9676.624	0.919	<0.001	<0.001	18.8/81.2
**C3**	**9267.967**	**9562.981**	**9394.599**	**0.94**	**<0.001**	**<0.001**	**81.2/15.8/3**
C4	9076.659	9471.867	9246.299	0.949	0.005	<0.001	1.3/15.6/2.2/80.8
C5	8981.748	9477.15	9194.495	0.938	0.06	<0.001	1.7/2/1.6/14.9/80

AIC, Akaike information criterion; BIC, Bayesian information criterion; aBIC, adjusted Bayesian information criterion; BLRT, the bootstrap likelihood ratio test; LMRT, Lo-Mendell-Rubin Likelihood Ratio Test.

### 3.3 Clusters of LCA models

**Figure 1:**
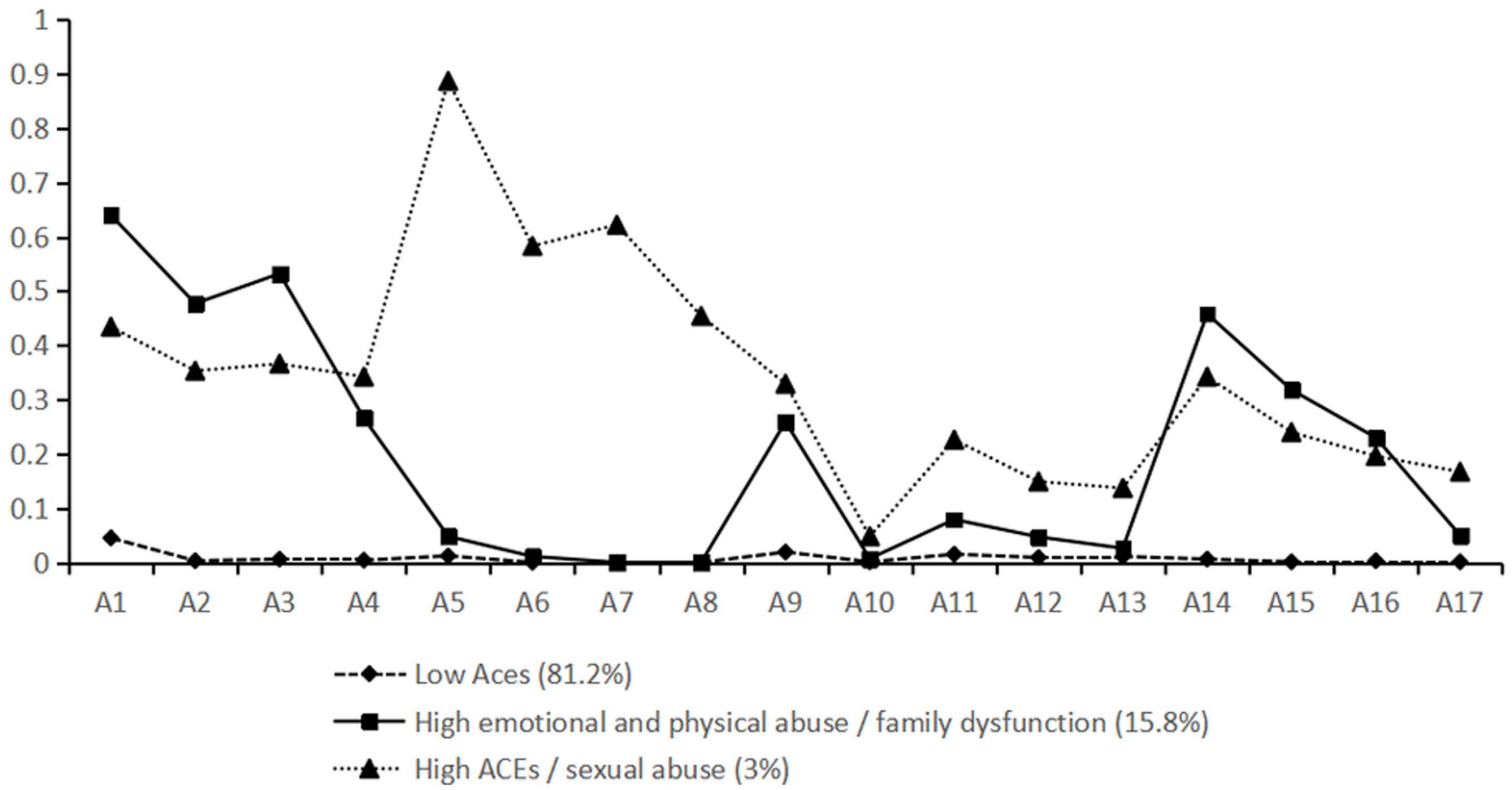
Response probabilities for LCA models A1, A2: emotional abuse, A3, A4: physical abuse, A5–A8: sexual abuse, A9, A10: household substance abuse, A11, A12: household mental illness, A13: household incarceration, A14: domestic violence, A15–A17: parental separation/divorce/death.

The probability of a latent class response is shown in Figure 1. (1) Low ACE cluster: This cluster had the lowest response rate among the three classes, except for emotional abuse (A1), which had a response rate of 4.5%; the other entries were close to 0. Therefore, in this cluster, respondents are unlikely to have experienced any ACEs. This cluster represented 82.2% of our overall sample. (2) High emotional and physical abuse/family dysfunction cluster: This cluster represented 15.8% of the total sample. These participants were more likely to have experienced emotional and physical abuse. They also had more serious problems with family functioning (i.e., parental separation/divorce/death, domestic violence, and household mental illness). (3) High ACE/sexual abuse cluster: Participants in this cluster had a high probability of having experienced all forms of childhood adversity, particularly sexual abuse, with a maximum response probability of nearly 90%. Details are shown in Figure 1.

### 3.4 Single factor analysis for three clusters

We found no significant differences between men and women in the three clusters (*p* = 0.709). Age, education, birthplace, physical illness, depression, and anxiety were significantly different in the three C clusters (*p* < 0.001). Details are shown in Table 3.

**Table 3:** Single factor analysis for three clusters.

Item	C1	C2	C3	X^2^	*p*
**Sex**				0.688	0.709
Male	708	136	23		
Female	861	169	35		
**Age (years)**				58.75	<0.001
18–30	518	68	20		
31–45	400	48	20		
46–60	376	90	6		
61–90	275	99	12		
**Education**				28.53	<0.001
High school or less	879	221	35		
More than high school	690	84	23		
**Birthplace**				28.26	<0.001
Rural	913	227	23		
Urban	656	78	35		
**Disease**				146.14	<0.001
No	1,150	132	39		
Hypertension	112	17	2		
Other diseases	297	156	17		
**Depression**				68.01	<0.001
No	775	97	18		
Mild depression	509	96	18		
Moderate or severe depression	285	112	22		
**Anxiety**				80.81	<0.001
No	925	117	18		
Mild anxiety	451	101	23		
Moderate or severe anxiety	193	87	17		

C1, Low ACE cluster; C2, High emotional and physical abuse/family dysfunction cluster; C3, High ACE/sexual abuse cluster.

### 3.5 Multinomial regression analysis for the three clusters

We used the statistically significant variables in the univariate analysis as independent variables, and the three clusters as dependent variables in multinomial logistic analysis. We should note that the C3 cluster had a relatively small number of samples, which may inevitably reduce the statistical effect. The results showed that age, education, place of birth, physical illness, anxiety, and depression were factors that influenced C2. Among them, ages 18–30 and 31–45 years as well as higher education level were protective factors in C2. Rural household registration, physical illness, anxiety, and moderate depression or above were risk factors in C2. In addition, 46–60 years, anxiety, and moderate depression or above were influencing factors in C3. Details are shown in Table 4.

**Table 4:** Multinomial regression analysis of different clusters using C1 as a reference.

Item	C2		C3	
	OR	Cl (95%)	OR	Cl (95%)
Demographic characteristics				
18–30 years	0.65*	0.41–1.01	1.04	0.42–2.54
31–45 years	0.48***	0.31–0.74	1.15	0.50–2.65
46–60 years	0.86	0.60–1.23	0.35*	0.13–0.97
61–90 years	1		1	
More than high school	0.75*	0.54–1.06	0.65	0.35–1.20
High school or less	1			
Rural	1.72***	1.28–2.31	0.93	0.53–1.61
Urban	1		1	
Physical health				
Other diseases	3.31***	2.45–4.47	1.55	0.81–2.96
Hypertension	0.79	0.44–1.42	0.49	0.11–2.24
No	1		1	
Mental health				
Moderate or severe depression	1.72*	0.99–3.00	3.26***	1.71–6.24
Mild depression	1.14	0.74–1.74	1.52	0.78–2.97
No	1		1	
Moderate or severe anxiety	2.19**	1.24–3.9	4.58*	1.36–15.38
Mild anxiety	1.46**	0.96–2.23	3.29*	1.29–8.39
No	1		1	

*p < 0.05, **p < 0.005, ***p < 0.001.

### 3.6 Depression and anxiety in different clusters

The results of ANOVA showed significant differences in depression and anxiety scores among the three clusters (*p* < 0.001). It can also be seen from the post-comparison that C2 and C3 showed higher anxiety and depression scores than C1. In other words, participants in C2 and C3 had a higher prevalence of anxiety and depression than C1. Details are shown in Table 5.

**Table 5:** Anxiety and depression scores in different clusters.

	C1 (M ± SD)	C2 (M ± SD)	C3 (M ± SD)	F	Comparison
Depression	0.63 ± 0.61	0.92 ± 0.64	0.95 ± 0.71	33.633***	C3 > C2 > C1
Anxiety	0.58 ± 0.64	0.91 ± 0.64	1 ± 0.75	42.388***	C3 > C2 > C1

***p < 0001.

## 4 Discussion

In the present study, we explored the clustering of ACEs among Chinese residents. The overall findings further confirm and extend existing evidence that ACEs profoundly impact physical and mental health in adulthood (38–41). Our results showed that 28.7% of Chinese residents have experienced at least one type of ACE, and 13.2% have experienced at least three or more types of ACE. The overall prevalence of ACEs is lower than those in other countries, with reported ACE prevalence in Kashmir and the United States of 88.2 and 60.9%, respectively (42, 43). The Dominican Republic reported an ACE prevalence of 80.6% (44). The prevalence of experiencing more than three ACEs in our study is consistent with the findings of a Brazilian study (45). Differences in regional environments (46) study populations, and measurement tools may explain these variations.

In addition, the lower prevalence of ACEs in China may be influenced by several specific cultural and social factors. Traditional Chinese family structures, which often emphasize collectivism and familial support, could play a protective role against certain types of childhood adversity. Additionally, China’s rapid economic development and improvement in living standards over recent decades may have contributed to a reduction in childhood adversities related to poverty, neglect, and household dysfunction. Moreover, the social policies promoting family stability and child protection implemented by the government might have further mitigated exposure to certain ACEs. However, while the prevalence of ACEs in China is lower, the findings still highlight significant public health challenges, particularly concerning the long-term mental and physical health of individuals affected by multiple ACEs. These results have broader implications for Chinese society, emphasizing the need for continued efforts to enhance mental health services, promote family education, and strengthen child protection policies. Understanding the unique clustering of ACEs in the Chinese context allows policymakers to tailor interventions that can address both current gaps in mental health support and future prevention strategies.

We identified three distinct clusters in the Chinese population, a low ACE cluster (C1, 81.2%), a high emotional and physical abuse/family dysfunction cluster (C2, 15.8%), and a high ACE/sexual abuse cluster (C3, 3%). The types and severity of adversity experienced by the participants in different clusters were not consistent. Most people belonged to the C1 group. These participants had little experience of childhood adversity. However, 3% of participants were likely to have experienced various forms of childhood adversity, particularly sexual abuse; the probability was relatively high for all four items regarding sexual abuse, as shown in Figure 1. These findings align with those of previous studies on Chinese university students (26), but our study’s broader demographic sample provides a more generalizable understanding of ACE clustering in the Chinese population.

We found significant associations between demographic factors, physical illness, mental health, and ACE clusters. Participants in the C3 cluster had a 3.26 times higher likelihood of moderate depression and a 4.58 times higher likelihood of anxiety compared to the C1 cluster.,. Similarly, the C2 group had a 1.72 and 2.19 times higher prevalence of depression and anxiety, respectively, compared with C1. That is, participants with depression and anxiety were more likely to have experienced different types of childhood adversity. These results underscore the long-term psychological impact of ACEs, as shown in previous multi-center studies (47). To address these risks, psychological counseling should be provided to high-risk groups, particularly those in the C2 and C3 clusters, to mitigate the adverse effects of ACEs.

Our study also showed that higher education was a protective factor for the C2 group. One explanation for this may be that the family environment of people with a higher education could be better, which could mean less likelihood of experiencing adversity in childhood. Previous research has also shown that early care and education programs can improve child maltreatment (48). Additionally, younger age groups (18–30 and 31–45 years) were less likely to belong to the C2 cluster compared to older adults (61–90 years), a finding supported by earlier studies (39). The geographic factor also played a role, with rural residents being more likely to belong to the C2 cluster, highlighting the need for geographically targeted public health interventions Studies have shown that children living in rural areas for a long period have more complex family relationships and lower utilization and availability of childcare resources by their parents, in comparison with children in urban areas. This can result in greater ACEs among rural children (49).

The C2 cluster also had a 3.31 times higher risk of physical diseases compared to the C1 group, particularly in areas like urinary, respiratory, digestive, and neurological health. These findings suggest that ACEs contribute to the risk of chronic diseases, though further research is needed to explore specific chronic conditions in greater detail. Unlike previous research, studies in Honduras and Brazil showed that women were at much higher risk of ACEs than men (50, 51). Women are a vulnerable group and are more likely to experience sexual abuse and domestic violence (52). However, in our study, we found that sex did not predict any ACE clustering. Liu et al. reached the same conclusion (53).

Finally, numerous previous studies have shown that the greater the adversity experienced in childhood, the higher an individual’s risk of anxiety and depression. However, it has been unclear whether this relationship can be found according to different clusters. Therefore, we compared depression and anxiety scores across clusters. Ultimately, it was found that clusters with greater childhood adversity scored higher, which further confirms that ACEs have a long-lasting effect on the mental health of the general population in China.

## 5 Strengths and limitations

This study has several strengths. First, most previous studies reporting the co-occurrence of ACEs in China were conducted among college students and adolescents. The co-occurrence of ACEs in the general population of China has not been reported. A comprehensive understanding of the clustering of ACEs at each stage is critical for policymakers and public health researchers. The findings of this study help to fill this evidence gap. Second, compared with previous studies, the present study provides more comprehensive evidence regarding the associations among various variables and clusters, including sociodemographic information, physical illness, and mental health.

However, several limitations should be noted. First, the retrospective nature of the study introduces the possibility of recall bias, as participants may not accurately remember events from their childhood. This may lead to either underreporting or overreporting of ACEs, which could affect the accuracy of our findings and potentially lead to biased estimates of the prevalence and impact of ACEs. Second, since this is a cross-sectional study, we cannot establish causal relationships between ACEs and health outcomes. Cross-sectional designs inherently limit the ability to determine the directionality of associations since both ACEs and mental health outcomes are measured simultaneously. This makes it difficult to discern whether ACEs lead to poor health outcomes or if pre-existing conditions influence the reporting of ACEs. Finally, this study does not assess the effectiveness of personal or group psychotherapy and counseling interventions in alleviating symptoms for individuals with ACEs. The inclusion of these factors in future studies would help provide a more comprehensive understanding of potential protective factors that may mitigate the negative effects of ACEs.

### 5.1 Implications for policy and practice

The findings of this study provide several actionable insights for policymakers and public health practitioners in addressing ACEs. First, based on the identified ACE clusters, tailored interventions can be designed for high-risk groups. Early detection and psychological counseling can be implemented through schools, healthcare systems, and community services. For early detection, validated tools such as the ACE Questionnaire, which assesses multiple forms of childhood trauma (e.g., emotional, physical, and sexual abuse, neglect, and household dysfunction), can be implemented in schools, pediatric clinics, and community health centers. These screening programs should be administered by trained staff during routine health assessments or school evaluations. Psychological counseling should include trauma-focused cognitive behavioral therapy (TF-CBT), which is effective in addressing the emotional and psychological impacts of ACEs. Additionally, group therapy programs providing peer support and trauma-informed care can offer further assistance. These interventions should be accessible through schools and community services, facilitated through partnerships between mental health professionals, educators, and healthcare providers. Second, rural residents are more likely to experience ACEs, highlighting the need for region-specific interventions. Expanding mobile mental health services, telemedicine programs, and community-based support groups can improve access to psychological counseling in rural areas. Resources such as mental health hotlines, educational outreach, and targeted awareness campaigns can further promote early intervention. These region-specific efforts aim to overcome the unique barriers to mental health care in rural populations. Third, integrating ACE screening into routine healthcare visits, particularly in pediatric settings, could help identify and support individuals at risk of long-term physical and mental health issues. Healthcare providers should be trained to administer the ACE Questionnaire and to refer at-risk individuals for appropriate counseling and support. Fourth, since higher education appears to be a protective factor against ACEs, policymakers should implement educational programs that promote resilience, parental education, and family support. These initiatives could include parental education on healthy parenting practices and school-based programs that teach children emotional regulation, conflict resolution, and coping skills. Finally, the high prevalence of sexual abuse in certain clusters emphasizes the need for stronger enforcement of child protection laws. Targeted initiatives could include awareness campaigns, legal reforms, and specialized training for law enforcement and social workers to reduce gender-based violence and abuse. These interventions, if implemented, can help mitigate the long-term impacts of ACEs and improve public health outcomes.

### 5.2 Implications for future research

The findings of this study open several avenues for future research, further studies should explore the relationship between ACEs and various chronic conditions, such as cardiovascular disease, kidney disease, diabetes, chronic pain, cancer, and dyslipidemia. Understanding the specific links between different ACE clusters and these health outcomes can provide more insight into the long-term physiological impacts of childhood trauma.

Additionally, longitudinal studies are essential to establish causal relationships between ACEs and chronic conditions. Future research could focus on following individuals over extended periods to assess how early exposure to ACEs influences the onset and progression of chronic diseases across the lifespan. Such studies should also examine protective factors, including social support, resilience, and access to mental health resources, which could help mitigate the long-term impacts of ACEs.

## 6 Conclusion

Our study identified clusters of childhood adversity among the ordinary residents of China. Three clusters were identified: a low ACE cluster, a high emotional and physical abuse/family dysfunction cluster, and a high ACE/sexual abuse cluster. It is important to note similarities and differences in the types and severity of childhood adversity experienced by people in these clusters. Policymakers should tailor public health strategies to the different clusters.

## Data Availability

The raw data supporting the conclusions of this article will be made available by the authors, without undue reservation.
